# Pathogenic role of the SP/ NK1R system in GBM cells through inhibiting the thioredoxin system

**DOI:** 10.22038/ijbms.2021.52902.11945

**Published:** 2021-04

**Authors:** Fatemeh Ghahremani, Reihaneh Sabbaghzadeh, Safieh Ebrahimi, Hosein Javid, Javad Ghahremani, Seyed Isaac Hashemy

**Affiliations:** 1 Department of Biology, Faculty of Science, Hakim Sabzevari University, Sabzevar, Iran; 2 Department of Clinical Biochemistry, Faculty of Medicine, Mashhad University of Medical Sciences, Mashhad, Iran; 3 Medical Laboratory Sciences Department, Varastegan Institute for Medical Sciences, Mashhad, Iran; 4 Department of Medicine, Faculty of Medicine, Shahid Beheshti University of Medical Sciences, Tehran, Iran; 5 Surgical Oncology Research Center, Mashhad University of Medical Sciences, Mashhad, Iran

**Keywords:** Glioblastoma multiforme, Neurokinin 1 receptor, Oxidative stress, Substance P, Thioredoxin

## Abstract

**Objective(s):**

Glioblastoma multiforme (GBM), a highly aggressive Grade IV brain tumor, is a significant public health issue due to its poor prognosis and incurability. Neuropeptide substance P (SP) plays a critical role in GBM tumor growth and development via activation of neurokinin-1receptor (NK1R). Moreover, SP is a pro-oxidant factor contributing to oxidative stress in various cell types. However, the link between SP and oxidative stress in cancer cells is not fully investigated. Here, we aimed to identify the effects of SP and NK1R antagonist, aprepitant, on the redox status of GBM cells.

**Materials and Methods:**

Resazurin assay was employed to determine the effect of aprepitant on viability of U87 glioblastoma cells. 2’,7’-dichlorodihydrofluorescein diacetate (H2DCFDA) assay was employed to measure the levels of intracellular reactive oxygen species (ROS). A quantitative real-time polymerase chain reaction was applied to measure the expression of proteins of the thioredoxin system. Commercial kits (ZellBio GmbH) were also used to measure the enzymatic activity of these proteins.

**Results:**

We found that SP increased ROS level in U87 GBM cells, and aprepitant significantly reduced this effect. Furthermore, we found that SP could also affect the thioredoxin system, a central antioxidant enzyme defense system. SP reduced both expression and enzymatic activity of the thioredoxin system’s proteins, Trx and thioredoxin reductase (TrxR) and these effects were significantly reduced by aprepitant.

**Conclusion:**

Our results indicated that SP activation of NK1R represented a link between oxidative stress and GBM and highlighted the need for further validations in future studies.

## Introduction

Glioblastoma multiforme (GBM), also referred to as a grade IV astrocytoma, arises from astrocytic glial cells responsible for supporting neuronal functions (1, 2). GBM is the most frequent and aggressive type of glial tumor associated with a low median survival of 14 months and increased resistance to chemo-radiotherapy (1, 2). Given the high morbidity and mortality rates and the low effectiveness of the currently used therapeutic strategies against GBM, there is a need for further etiological studies focusing on the elucidation of the molecular mechanisms involved in GBM pathogenesis. Recently, the neuropeptides present in the tumor microenvironment have gained considerable attention due to their tumor-promoting functions (3, 4). Neuropeptide substance P (SP) is one of tachykinin family members and broadly available throughout the CNS (5). SP-induced biological responses are mediated via activation of neurokinin-1 receptor (NK1R), a member of the neurokinin G protein-coupled receptors (GPCRs) family (6). SP activation of NK1R regulates many pathophysiological functions, including transmission of pain information, chemotherapy-induced nausea and vomiting (CINV), neurogenic inflammatory reactions, and induction of various tumor-promoting responses (6-10). Remarkably, the over-expression of NK1R has been reported in different tumor types, including glioblastomas associated with more aggressive behavior of the tumor (11-15). For instance, Hennig *et al.* reported that approximately 100% of all cases of glioblastomas express NK1R, while 75% of cases of astrocytoma express NK1R. Thus, the NK1R density level is related to the degree of malignancy (e.g., glioblastomas express more NK1R than do astrocytomas) (16).

Aprepitant is a specific NK1R antagonist approved by the US FDA to prevent CINV (17), and no markedly unwanted side effects have been found for this antagonist so far (18). Furthermore, some investigations focused on the apoptotic properties and anticancer activities of this compound on various cancer cell lines from human or animal tissues (19, 20).

SP activation of NK1R has been shown to induce glioblastoma cell proliferation through activation of Erk/MAPK and Akt pathways (21). SP/NK1R signaling also triggers inflammatory responses by increasing pro-inflammatory cytokines involved in gliomas growth and development (21). It has been suggested that the SP/NK1R system is of importance in modifying the cellular redox environment. SP has been shown to affect the redox balance of the body by augmenting the reactive oxygen species (ROS) production or reducing antioxidant defense enzymes’ expression and further inducing oxidative stress (22, 23). The pathological condition of various clinical disorders is exacerbated by oxidative stress responses mediated by SP/NK1R signaling. However, the effects of SP/NK1R signaling on the redox status of tumor cells have not been thoroughly investigated (24, 25). Since oxidative stress has a critical role in glioblastoma initiation and progression, revealing the relationship between SP/NK1R signaling and GBM redox status is essential for developing more effective therapeutic approaches for GBM patients. 

Here we explored the effect of exogenous SP on ROS and the thioredoxin system, one of the main cellular redox buffers in the GBM cell line. we also investigated the modulatory effect of a commercially available NK-1R antagonist, aprepitant, on SP-induced alteration of the redox system.

## Materials and Methods

***Cell culture***

Experiments were carried out with U87 human primary glioblastoma cell line. The cells were obtained from the National Cell Bank of Iran (Pasteur Institute, Iran, Tehran). These cells were grown in RPMI 1640 and Ham’s F-12 and supplemented with 10% fetal bovine serum (FBS) and 1% antibiotics, penicillin-streptomycin, (all products were from Gibco BRL). SP and aprepitant were obtained from Sigma-Aldrich (US life science company).

***Resazurin-based viability assay***

Resazurin-based assay, a simple and sensitive method for measuring cellular viability, is based on reduction of nonfluorescent dye resazurin to the strongly-fluorescent dye resorufin through the activity of dehydrogenase enzymes in metabolically active cells (26). The fluorescence output is proportional to the number of viable cells that are available in a sample. In short, 2.5 × 10^4^ U87 glioblastoma cells were cultured in 96-well plates and treated with various concentrations of aprepitant 0 (control), 5, 10, 25, 35, and 50 μM for 24 hr. Subsequently, the medium was discarded, and to each well was added 10 μl resazurin solution (0.01 mg/mL dissolved in phosphate-buffered saline), and the wells were incubated at 37 °C for 3 hr. The sample’s fluorescence intensity was measured using a fluorescence spectrometer at wavelengths of 570 nm and 600 nm. The obtained values were changed to percentage survival rates by comparing the absorbance of cells (treated vs untreated), and the 50% inhibitory concentration (IC_50_) value was calculated by the statistical software, GraphPad Prism 6.

***Measurement of reactive oxygen species content***

To measure the levels of the intracellular ROS, 2’,7’-dichlorodihydrofluorescein diacetate (H2DCFDA) assay was employed. H2DCFDA passes cell membrane and is cleaved by cellular esterase to create nonfluorescent 2′,7′-dichlorodihydrofluorescein (H2DCF), H2DCF is then oxidized to highly fluorescent 2′,7′-dichlorofluorescein (DCF) by ROS. In short, 75 × 10^4^ cells were seeded in a 6-well culture plate for 24 hr. Subsequently, cells were incubated at 37 °C for 30 min with 10 μM DCFHDA before treatment. After this, U87 glioblastoma cells were exposed to SP (100 and 400 nM) alone or in combination with aprepitant (15 μM) for another 24 hr. Tertbutyl Hydrogen Peroxide (TBHP) (50 mM) was selected as a positive control. The fluorescence intensity of DCF is then measured using a Perkin-Elmer atomic absorption spectrophotometer at excitation and emission wavelengths of 495nm and 529 nm, respectively.

***RNA isolation and quantitative real-time PCR (qRT-PCR)***

The total RNA was extracted from the U87 cell line with the FavorPrep blood/ cultured cell total RNA mini kit (Yekta Tajhiz, Iran) according to the manufacturer’s instruction. A nanodrop spectrophotometer and agarose gel electrophoresis were used to measure the concentration and purity of the extracted RNA. Complementary DNA (cDNA) is synthesized from reverse transcription of the sample RNA using cDNA Synthesis Kit (Pars Tous biotechnology, Iran) as instructed. qRT-PCR amplifications were carried out in the Roche LightCycler 96 using SYBR Green qPCR Master Mix (No ROX) (Ampliqon, Denmark). Glyceraldehyde-3-phosphate dehydrogenase (GAPDH), a housekeeping gene, was also used as an internal reference gene, and the relative gene expression level was computed using the 2^_DDCT^ method.

***Assessment of thioredoxin (Trx) and thioredoxin reductase (TrxR) activity in vitro***

Activities of thioredoxin (Trx) and thioredoxin reductase (TrxR) were measured separately with relevant commercial kits (ZellBio GmbH, Germany). ZellBio GmbH kits employ a quantitative sandwich enzyme-linked immunosorbent assay (ELISA) to assay Trx and TrxR activity. The experiment was conducted according to the instructions of the manufacturer. The activity of Trx and TrxR was evaluated in U/L and µM/min/mL, respectively, according to the manufacturer’s instructions.

***Statistical analysis***

All experiments were carried out separately at least 3 times, and the results are presented as mean±standard deviation (SD) (n= 3). Statistical analyses were performed by using GraphPad Prism 6 statistical software. Bonferroni *post hoc* analysis t-test was applied to assess multi-group comparisons following ANOVA. The *P*-value of less than 0.05 was considered statistically significant.

## Results

***Results of the cell viability***

**Figure 1:**
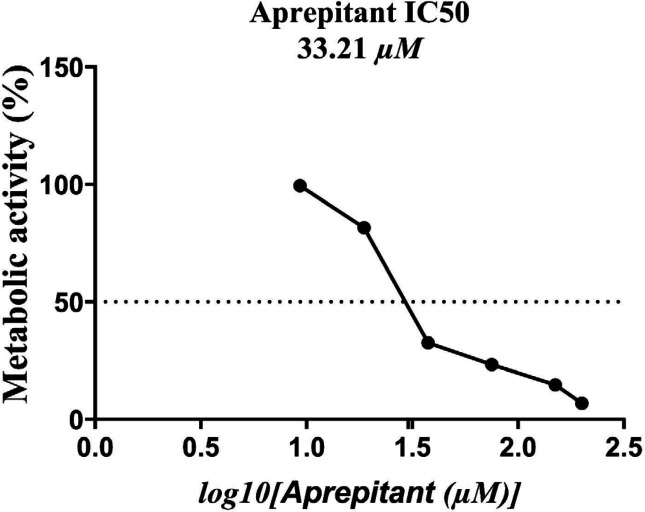
Results of the resazurin-based cell viability at increasing concentrations of aprepitant after 24 hr. The IC_50 _value of aprepitant in U87 glioblastoma cells was about 33.21 μM

The results of the resazurin-based cell viability assay at increasing concentrations of aprepitant are illustrated in Figure 1. Aprepitant caused a dose-dependent decrease in U87 glioblastoma cell viability and metabolic activity. As shown in Figure 1, the exposure of U87 cells to aprepitant after 24 hr resulted in a significant reduction in cells’ metabolic activity with an estimated IC_50_ value of around 33.21 μM. Furthermore, the concentration of 15 μM was selected as the experimental concentration regarding the dose-dependent changes in cell viability.

***Effects of SP and aprepitant on intracellular ROS levels in U87 glioblastoma cells***

SP has been found as a pro-oxidant factor inducing ROS overproduction in various cell types, including immune-inflammatory cells (27, 28), epithelial cells (23, 29), and sensory neurons (30) through activation of NK1R. However, ROS-inducing effects of SP in glioblastoma tumor cells are unclear. Hence, to determine the SP/NK1R signaling effect in U87 glioblastoma cells, we assessed the intracellular ROS levels in response to SP (100 and 400 nM) alone or in combination with aprepitant (15 μM) by DCFH-DA assay.

**Figure 2:**
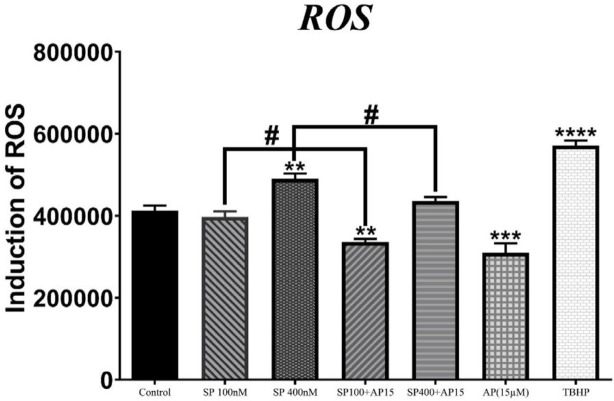
Effects of SP and aprepitant on intracellular ROS generation. U87 glioblastoma cells were treated with the preferred concentration of SP (100 and 400 nM) alone or in combination with aprepitant (15 μM) for 24 hr, and ROS generation was evaluated by DCFH-DA assay. The results indicate that intracellular ROS generation was significantly reduced in cells treated with aprepitant with or without pretreatment with SP

As shown in Figure 2, SP increased ROS levels in U87 glioblastoma cells; however, meaningful change was observed when cells were exposed to SP 400 nM. Moreover, intracellular ROS level was significantly reduced in cells treated for 24 hR with aprepitant (15 μM) with or without SP (100 and 400 nM) pretreatment. These results suggested that SP/NK1R signaling affects the GBM cellular redox state partly by altering the intracellular ROS generation.

***Effects of SP and aprepitant on the mRNA expression of thioredoxin in U87 glioblastoma cells***

**Figure 3:**
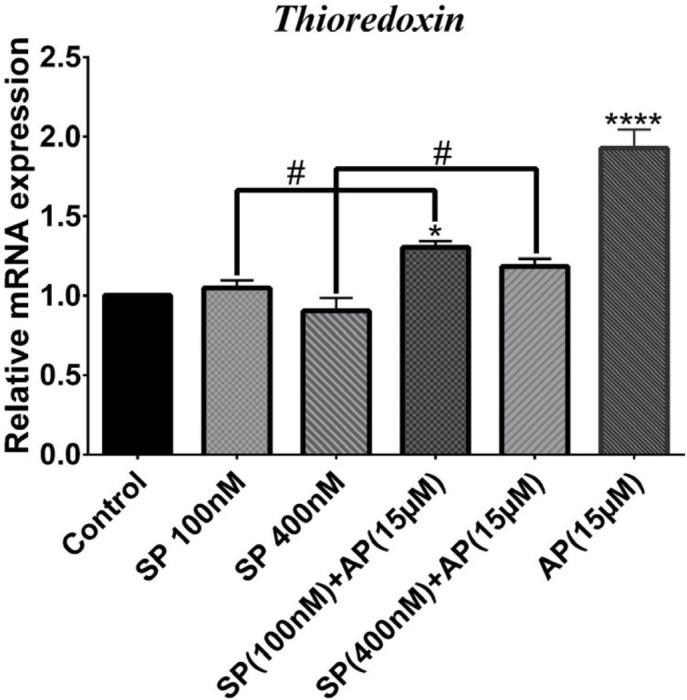
SP and aprepitant's effects on mRNA expression level of thioredoxin (Trx) in U87 glioblastoma cells. The results indicate that mRNA expression of Trx is significantly elevated in cells treated with aprepitant (15 μM) with or without pretreatment with SP (100 and 400 nM) compared to the untreated control cells. The levels of expression of Trx was normalized by GAPDH mRNA levels and presented as a mean±SD (*P*<0.05)

Thioredoxin system is one of the most crucial antioxidative defense systems in the living cell composed of the enzymatic proteins Trx and TrxR as well as NADPH (31, 32). To further clarify SP/NK1R system’s prooxidative effects in GBM cells, we also explored the alteration of the mRNA expression level of Trx in response to SP (100 and 400 nM) alone or in combination with aprepitant (15 μM) by q RT-PCR. As shown in Figure 3, effects of SP on the expression of Trx were not significant. However, the expression of Trx was significantly increased in cells treated for 24 hr with aprepitant (15 μM) with or without SP (100 and 400 nM) pretreatment. 

***Effects of SP and aprepitant on enzymatic activity of the proteins of the thioredoxin system in U87 glioblastoma cells***

**Figure 4:**
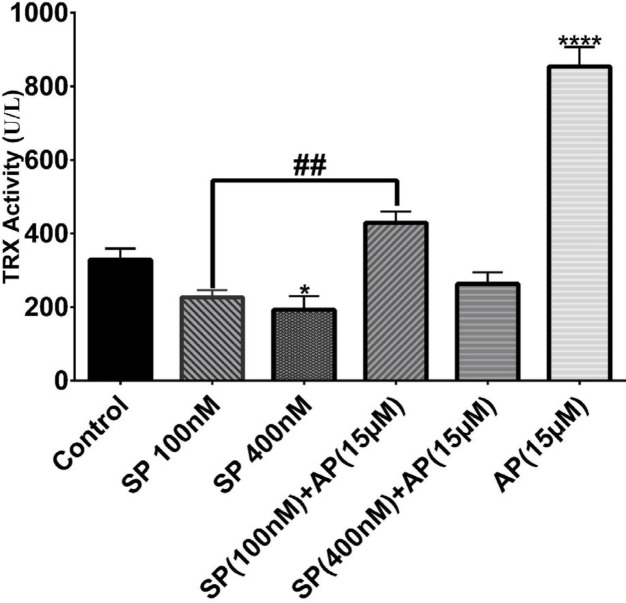
Effects of SP and aprepitant on thioredoxin (Trx) activity in U87 glioblastoma cells. The results indicate that Trx activity is significantly elevated in cells treated for 24 hr with aprepitant (15 μM) with or without SP (100 and 400 nM) pretreatment compared with the untreated control cells. The activity of Trx is expressed as U/l

**Figure 5:**
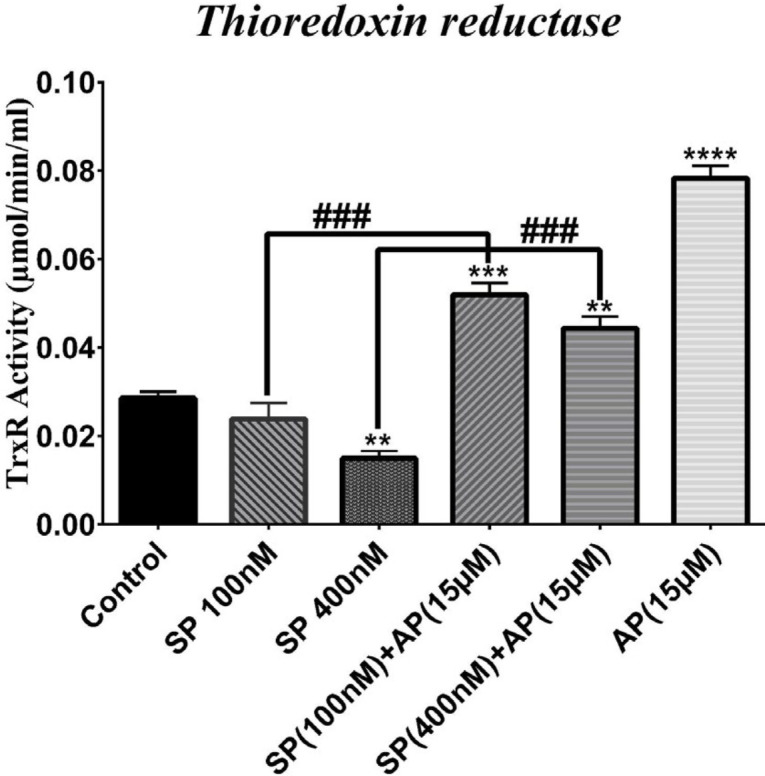
Effects of SP and aprepitant on thioredoxin reductase (TrxR) activity in U87 glioblastoma cells. The results indicate that TrxR activity is significantly elevated in cells treated for 24 hr with aprepitant (15 μM) with or without SP (100 and 400 nM) pretreatment compared with the untreated control cells. The activity of TrxR is expressed as µM/min/ml

To further support the observed effects of SP and aprepitant on the thioredoxin system, we also measured the activity of both Trx and TrxR. We assessed the activity of Trx and TrxR in response to SP (100 and 400 nM) alone or in combination with aprepitant (15 μM). As shown in Figures 4 and 5, SP reduced the activity of both Trx and TrxR in U87 glioblastoma cells; however, meaningful changes were observed when cells were exposed to SP (400 nM). Moreover, the activity of both Trx and TrxR was significantly increased in cells treated for 24 hr with aprepitant (15 μM) with or without SP (100 and 400 nM) pretreatment. 

SP: substance P; ROS:reactive oxygen species

SP: substance P

SP: substance P

SP: substance P

## Discussion

In the current study, we examined the effects of exogenous SP on the redox status of GBM *in vitro*. Our results indicate that exogenous SP increased ROS generation and reduced both expression and enzymatic activity of the Trx system’s proteins, and NK1R antagonist, aprepitant significantly decreased these effects. 

Oxidative stress is caused by oxidant-antioxidant imbalance, leading to excessive generation of ROS (33). ROS molecules including superoxide anion (O_2_^•−^), hydrogen peroxide (H_2_O_2_), and hydroxyl radical (•OH) are extremely reactive and can cause detrimental effects on proteins, carbohydrates, nucleic acids, and lipids (34). 

Although several lines of evidence have shown that mitochondrial ROS accumulation has a critical role in apoptotic cell death during physiologic and pathologic conditions (35-37), some researchers have recently shown that intracellular ROS production induces the proinflammatory processes, resulting in cancer cell progression and development (38-40). Thus, finding potent, safe, and effective antioxidants has attracted tremendous attention (41). These findings, combined with our previous results indicate that aprepitant could enhance anticancer activity by suppressing inflammatory-regulated ROS generation (9). 

In this context, similar to our study, aprepitant treatment has been shown to inhibit ROS production in arthritis fibroblast-like synoviocytes (24), inflammatory macrophages (42), and esophageal squamous cell carcinoma cells (9). In contrast, administration of aprepitant has been reported to increase ROS generation in acute myeloid leukemia (AML) cells (43, 44) and triple-negative breast cancer (TNBC) (41) and further increasing the sensitivity to chemotherapy. This disparity can be accounted for by the use of different dosages of SP and aprepitant and the exposure duration, differences in oxidative capacities of cancer cells, and the use of different redox regulating signaling pathways by different cell types. Given that ROS initiates both pro-apoptotic and pro-survival effects depending on intensity and exposure time (45), it can therefore be concluded that aprepitant might affect the dual functions of ROS regarding the applied dose.

Notably, the brain is more vulnerable to ROS-mediated damage, mainly due to high cerebral metabolic rate and a relatively low cellular regenerative capacity (46-48). Oxidative damages of these macromolecules ultimately promote GBM tumor initiation and progression via interfering in critical cellular processes, including cell proliferation, apoptosis, migration, and resistance (34). Accordingly, our finding that SP increases ROS generation in glioblastoma cells, which was inhibited by aprepitant, suggests a possible role for the SP/NK1R signaling in GBM pathogenesis through oxidative stress. Similar to our finding in GBM cells, an increase in ROS generation in response to SP has also been reported in the immune-inflammatory cells (27, 28), gastric and respiratory tract epithelial cells (23, 29), and sensory neurons (30). In gastric epithelial cells, SP activation of NK1R promotes the development of hemorrhagic lesions of the stomach wall through increased cytotoxic ROS formation (23). SP activates neutrophil NADPH oxidase, one of ROS’s main sources in cells, through NK1R to generate cytotoxic ROS, leading to bladder hyperactivity (49-51). Importantly, SP has been shown to activate microglial NADPH oxidase to produce ROS (52). Given the abundance of microglial cell populations in GBM, it is likely that activation of microglial NADPH oxidase might be a possible mechanism by which SP induces ROS generation in GBM cells. However, further validations in future studies are required to elucidate the molecular mechanism involved in ROS-inducing effects of SP in GBM. To further clarify SP’s prooxidative effects in GBM cells, we also explored the alteration of antioxidant status in response to SP. To overcome oxidative stress, cells are equipped with nonenzymatic as well as enzymatic (superoxide dismutase (SOD), catalase, glutathione peroxidase (GPx), and the thioredoxin system) antioxidant defense mechanisms (53). The Trx system is one of the most crucial antioxidative defense systems in the living cell composed of Trx, TrxR, and an electron donor, NADPH (31, 54). Trx is a small thiol protein of approximately 12 kD containing redox-active cysteine residues within its conserved active site (Cys-Gly-Pro-Cys) that interact with the thiol groups on proteins to create a disulfide bond and further reducing target proteins. TrxR is an NADPH-dependent oxidoreductase that returns Trx to its reduced form and plays a crucial role in preserving Trx turnover from the oxidized to the active and reduced state (55). Trx induces neuroprotective properties via modulating oxidative stress, and Trx overexpression has been shown as an effective approach against neurotoxicity. In this line, Hattori *et al.* demonstrated that intravenous injection of recombinant hTrx in mice significantly reduces brain damage following transient focal cerebral ischemia (56). Over-expression of Trx was reported to protect against oxidative stress-induced neurodegeneration of retinal ganglion cells (57). Over-expression of mitochondrial Trx also protects against tertbutyl hydroperoxide-induced apoptosis of human neuroblastoma cells (58). Here, we indicate that in GBM cells treated with SP, ROS generation is increased. Simultaneously, both expression and enzymatic activity of the Trx system proteins are reduced, highlighting the importance of this system in controlling ROS levels in GBM cells. Furthermore, we found that aprepitant significantly inhibits SP-mediated alteration of the Trx system. 

Many other studies have also explored the oxidative stress inhibitory effects of several NK1R antagonists in various clinical disorders, which result in a significant reduction in ROS levels and elevation of antioxidant enzymes, thereby leading to disease improvement. From this point of view, our study could also display a neuroprotective role for aprepitant in GBM cells by inhibiting SP’s prooxidative effects in GBM cells either by reducing ROS generation or increasing the thioredoxin antioxidant defense mechanisms. However, the Trx system proteins’ overexpression in GBM has been shown to induce intrinsic chemoresistance of GBM cells to cytostatic drugs (59). Thus, different Trx system properties need to be taken into account when considering aprepitant as a therapeutic drug in GBM.

## Conclusion

Here we report that SP activation of NK1R represents a link between oxidative stress and cancer. The blockage of NK1R with aprepitant effectively reduces oxidative stress in GBM suggesting the clinical significance of aprepitant in stress-related cancer including GBM. However, further *in vitro* and *in vivo* experiments should be performed to elucidate the redox modifying functions of SP/NK1R signaling and their clinical significance in GBM cells.
